# Supplementary material for: The High Prevalence of Sarcopenia in Rheumatoid Arthritis in the Korean Population: A Nationwide Cross-Sectional Study
Source: Healthcare (Basel). 2023 May 12;11(10):1401. doi: 10.3390/healthcare11101401 (PMC10218272; doi:10.3390/healthcare11101401)
Supplement: Supplementary file 1 [file healthcare-11-01401-s001.zip › healthcare-2306559-supplementary.pdf]

Supplement Table S1. Baseline characteristics of the participants with and without rheumatoid arthritis in group with age < 40 years

	**Men** (*n* = 2399)					**Women** (*n* = 3239)				
	**RA** (*n* = 1)		**Non-RA** (*n* = 2398)			**RA** (*n* = 11)		**Non-RA** (*n* = 3228)		
	**n**	**%**	**n**	**%**	***p***	**n**	**%**	**n**	**%**	***p***
**Age (in years)**	32		30.91 ± 5.855		0.852	31.64 ± 5.870		30.84 ± 5.898		0.654
**Obesity**					0.187					0.720
Underweight	0	0	90	3.8		1	9.1	367	11.4	
Normal weight	0	0	1469	61.3		9	81.8	2319	71.8	
Overweight	1	100.0	839	35.0		1	9.1	542	16.8	
**Menopause**					-					0.869
Yes	-	-	-	-		0	0	8	0.2	
No	-	-	-	-		11	100	3220	99.8	
**Hypertension**					0.111					0.142
Normal	0	0	1179	49.2		11	100	2697	83.6	
Pre-hypertension	0	0	838	34.9		0	0	431	13.4	
Hypertension	1	100.0	381	15.9		0	0	100	3.1	
**Diabetes mellitus**					0.686					0.889
Normal	1	100.0	2061	85.9		10	90.9	2952	91.4	
Impaired fasting glucose	0	0	293	12.2		0	0	227	7.0	
Diabetes mellitus	0	0	44	1.8		1	9.1	49	1.5	
**Dyslipidemia**					0.377					0.226
Normal	0	0	1052	43.9		3	27.3	1468	45.5	
Dyslipidemia	1	100.0	1346	56.1		8	72.7	1760	54.5	
**Alcohol consumption**					0.138					0.208
None	0	0	435	18.1		3	27.3	1527	47.3	
Moderate	0	0	1387	57.8		7	63.6	1470	45.5	
Heavy	1	100.0	576	24.0		1	9.1	49	7.2	
**Smoking status**					0.594					0.421
Never	0	0	600	25.0		10	90.9	2659	82.4	
Past	1	100.0	528	22.0		1	9.1	295	9.1	
Current	0	0	1270	53.0		0	0	274	8.5	

**Household income**					0.289					0.777
Lowest	0	0	190	7.9		1	9.1	240	7.4	
Lower middle	1	100.0	617	25.7		3	27.3	849	26.3	
Upper middle	0	0	854	35.6		4	36.4	1135	35.2	
Highest	0	0	737	30.7		3	27.3	1004	31.1	
**Education**					0.322					0.694
Primary school or lower	0	0	16	0.7		0	0	23	0.7	
Middle school	0	0	48	2.0		0	0	67	2.1	
High school	0	0	1140	47.5		5	45.5	1531	47.4	
University or higher	1	100.0	1194	49.8		6	54.5	1607	49.8	
**Sarcopenia**					0.153					0.713
Absent	0	0	2031	84.7		10	90.9	2743	85.0	
Present	1	100.0	367	15.3		1	9.1	485	15.0	

RA = rheumatoid arthritis.

Data are given as mean ± standard deviation or the number with percentage.

Supplement Table S2. Baseline characteristics of the participants with and without rheumatoid arthritis in group with 40 ≤ age < 59 years

	Men (n = 2802)					Women (n = 3760)				
	RA (n = 4)		Non-RA (n = 2798)			RA (n = 31)		Non-RA (n = 3729)		
	n	%	n	%	p	n	%	n	%	p
**Age (in years)**	52.50 ± 2.646		49.08 ± 5.811		0.239	52.06 ± 4.434		49.12 ± 5.645		0.004
**Obesity**					0.162					0.191
Underweight	0	0	51	1.8		2	6.5	85	2.3	
Normal weight	1	25.0	1610	57.5		22	71.0	2485	66.6	
Overweight	3	75.0	1137	40.6		7	22.6	1159	31.1	
**Menopause**					-					< 0.001
Yes	-	-	-	-		25	80.6	1535	41.2	
No	-	-	-	-		6	19.4	2194	58.8	
**Hypertension**					0.274					0.009
Normal	1	25.0	900	32.2		7	22.6	1880	50.4	
Pre-hypertension	0	0	843	30.1		14	45.2	949	25.4	
Hypertension	3	75.0	1055	37.7		10	32.3	900	24.1	
**Diabetes mellitus**					0.571					0.215
Normal	2	50.0	1683	60.2		27	87.1	2833	76.0	
Impaired fasting glucose	1	25.0	787	28.1		1	3.2	663	17.8	
Diabetes mellitus	1	25.0	328	11.7		3	9.7	233	6.2	
**Dyslipidemia**					0.925					0.922
Normal	1	25.0	758	27.1		7	22.6	870	23.3	
Dyslipidemia	3	75.0	2040	72.9		24	77.4	2859	76.7	
**Alcohol consumption**					0.418					0.127
None	2	50.0	615	22.0		22	71.0	2180	58.5	
Moderate	1	25.0	1467	52.4		9	29.0	1390	37.3	
Heavy	1	25.0	716	25.6		0	0	159	4.3	
**Smoking status**					0.407					0.054
Never	1	25.0	451	16.1		26	83.9	3469	93.0	
Past	2	50.0	1074	38.4		3	9.7	94	2.5	
Current	1	25.0	1273	45.5		2	6.5	166	4.5	

**Household income**					0.245					0.036
Lowest	0	0	246	8.8		6	19.4	433	23.0	
Lower middle	1	25.0	610	21.8		9	29.0	913	18.7	
Upper middle	0	0	900	32.2		11	35.5	1115	39.5	
Highest	3	75.0	1042	37.2		5	16.1	1268	34.0	
**Education**					0.390					< 0.001
Primary school or lower	1	25.0	322	11.9		14	45.2	856	23.0	
Middle school	1	25.0	422	15.1		10	32.3	697	18.7	
High school	1	25.0	1041	37.2		5	16.1	1474	39.5	
University or higher	1	25.0	1003	35.8		2	6.5	702	18.8	
**Sarcopenia**					0.849					0.039
Absent	3	75.0	2207	78.9		18	58.1	2772	74.3	
Present	1	25.0	591	21.1		13	41.9	957	25.7	

RA = rheumatoid arthritis.

Data are given as mean ± standard deviation or the number with percentage.

Supplement Table S3. Baseline characteristics of the participants with and without rheumatoid arthritis in group with age ≥ 60 years

	**Men** (*n* = 2188)					**Women** (*n* = 2799)				
	**RA** (*n* = 21)		**Non-RA** (*n* = 2167)			**RA** (*n* = 88)		**Non-RA** (*n* = 2711)		
	**n**	%	**n**	%	*p*	**n**	%	**n**	%	*p*
**Age (in years)**	72.10 ± 7.569		68.75 ± 6.088		0.013	69.17 ± 6.837		69.10 ± 6.289		0.914
**Obesity**					0.097					0.022
Underweight	0	0	100	4.6		4	4.5	82	3.0	
Normal weight	12	57.1	1455	67.1		60	68.2	1571	57.9	
Overweight	9	42.9	612	28.2		24	27.3	1058	39.0	
**Menopause**					-					0.184
Yes	-	-	-	-		80	90.9	2566	94.3	
No	-	-	-	-		8	9.1	155	5.7	
**Hypertension**					0.392					0.866
Normal	7	33.3	405	18.7		14	15.9	452	16.7	
Pre-hypertension	3	14.3	539	24.9		21	23.9	587	21.7	
Hypertension	11	52.4	1223	56.4		53	60.2	1672	61.7	
**Diabetes mellitus**					0.709					0.709
Normal	12	57.1	1123	51.8		53	60.2	1601	59.1	
Impaired fasting glucose	1	4.8	603	27.8		20	22.7	574	21.2	
Diabetes mellitus	8	38.1	441	20.4		15	17.0	536	19.8	
**Dyslipidemia**					0.233					0.020
Normal	9	42.9	667	30.8		15	17.0	259	9.6	
Dyslipidemia	12	57.1	1500	69.2		73	83.0	2452	90.4	
**Alcohol consumption**					0.004					0.145
None	14	66.7	807	37.2		76	86.4	2174	80.2	
Moderate	7	33.3	1127	52.0		12	13.6	518	19.1	
Heavy	0	0	233	10.8		0	0	19	0.7	
**Smoking status**					0.609					0.255
Never	3	14.3	355	16.4		77	87.5	2464	90.9	
Past	11	52.4	1201	55.4		4	4.5	129	4.8	
Current	7	33.3	611	28.2		7	8.0	118	4.4	

**Household income**					0.008					0.271
Lowest	15	71.4	846	39.0		50	56.8	1347	49.7	
Lower middle	3	14.3	621	28.7		18	20.5	244	25.4	
Upper middle	1	4.8	387	17.9		11	12.5	233	14.1	
Highest	2	9.5	313	14.4		9	10.2	62	10.8	
**Education**					0.145					0.182
Primary school or lower	12	57.1	967	44.6		75	85.2	2172	80.1	
Middle school	4	19.0	443	20.4		9	10.2	244	9.0	
High school	5	23.8	477	22.0		3	3.4	233	8.6	
University or higher	0	0	280	12.9		1	1.1	62	2.3	
**Sarcopenia**					0.001					0.462
Absent	7	33.3	1445	66.7		60	68.2	1745	64.4	
Present	14	66.7	722	33.3		28	31.8	966	35.6	

RA = rheumatoid arthritis.

Data are given as mean ± standard deviation or the number with percentage.
